# Mitochondrial Dysfunction Is a Common Denominator Linking Skeletal Muscle Wasting Due to Disease, Aging, and Prolonged Inactivity

**DOI:** 10.3390/antiox10040588

**Published:** 2021-04-11

**Authors:** Hayden W. Hyatt, Scott K. Powers

**Affiliations:** Department of Applied Physiology and Kinesiology, University of Florida, Gainesville, FL 32611, USA; spowers@hhp.ufl.edu

**Keywords:** oxidative stress, reactive oxygen species, muscle atrophy, calpain, protein synthesis, proteolysis

## Abstract

Skeletal muscle is the most abundant tissue in the body and is required for numerous vital functions, including breathing and locomotion. Notably, deterioration of skeletal muscle mass is also highly correlated to mortality in patients suffering from chronic diseases (e.g., cancer). Numerous conditions can promote skeletal muscle wasting, including several chronic diseases, cancer chemotherapy, aging, and prolonged inactivity. Although the mechanisms responsible for this loss of muscle mass is multifactorial, mitochondrial dysfunction is predicted to be a major contributor to muscle wasting in various conditions. This systematic review will highlight the biochemical pathways that have been shown to link mitochondrial dysfunction to skeletal muscle wasting. Importantly, we will discuss the experimental evidence that connects mitochondrial dysfunction to muscle wasting in specific diseases (i.e., cancer and sepsis), aging, cancer chemotherapy, and prolonged muscle inactivity (e.g., limb immobilization). Finally, in hopes of stimulating future research, we conclude with a discussion of important future directions for research in the field of muscle wasting.

## 1. Introduction

In healthy adults, skeletal muscles comprise 40%–50% of total body mass; muscles provide several vital physiological functions and are required for both locomotion and breathing. Notably, muscle fibers are also an endocrine organ and play a key role in glucose homeostasis [1,2]. The loss of skeletal muscle mass due to disease or other conditions not only reduces an individual’s quality of life but is also associated with increased morbidity and mortality [3,4]. Numerous causes of skeletal muscle wasting exist, including disease (e.g., cancer, sepsis, etc.), cancer chemotherapy (e.g., doxorubicin), aging, and extended durations of muscle inactivity (e.g., limb immobilization). Although the regulation of muscle mass is a multifactorial process, studies have identified common elements that contribute to skeletal muscle atrophy across several diseases and conditions [5]. For example, growing evidence suggests that mitochondrial dysfunction is a common denominator that contributes to muscle loss during numerous diseases, aging, and prolonged periods of inactivity. This review discusses the cell signaling pathways that connect mitochondrial dysfunction and muscle wasting. Specifically, this report will debate the strength of the experimental evidence that directly links mitochondrial dysfunction to muscle wasting in response to prolonged inactivity, aging, chemotherapy agents (i.e., doxorubicin), and specific diseases (i.e., cancer and sepsis). We begin with an overview of the cellular events leading to skeletal muscle atrophy.

### Primer on Skeletal Muscle Wasting

Skeletal muscle mass is regulated by the balance of the rates of protein synthesis and protein breakdown. A detailed discussion of the control of muscle protein synthesis and proteolysis is outside the scope of this review, and the reader is directed to comprehensive reviews for more information [5,6,7]. Nonetheless, to provide context for readers new to the field of skeletal muscle wasting, we provide a short summary of the key events that regulate skeletal muscle mass.

Muscle protein synthesis is controlled by the complex interplay between transcription and translational events [8]. While mRNA is an essential precursor for protein synthesis, differences exist between the abundance of mRNA and their respective protein; indeed, only 40% of cellular proteins are highly correlated with the abundance of the corresponding mRNA [9,10]. This finding indicates that translational efficacy plays a major role in the control of protein synthesis. While the majority of investigations have focused on the importance of the Akt/mechanistic target of rapamycin (mTOR) pathway in the regulation of protein synthesis, growing evidence reveals that muscle protein synthesis can be regulated by mTOR independent mechanisms (reviewed in [11]). However, at present, the components of the mTOR independent pathways responsible for the control of protein synthesis remain unknown.

Increased mechanical load on skeletal muscle fibers promotes an increase in muscle protein synthesis and results in fiber hypertrophy [11]. In contrast, prolonged muscle inactivity and/or increased production of reactive oxygen species (ROS) in muscle fibers depresses protein synthesis and fiber atrophy ensues [12,13,14]. The mechanism(s) to explain oxidative stress-induced depression of muscle protein synthesis is hypothesized to result from depressed anabolic signaling, leading to decreased translation [15].

Skeletal muscle protein degradation results from the coordinated action of four proteolytic systems: (1) autophagy; (2) the ubiquitin–proteasome system; (3) calpains; and (4) caspase-3. Numerous detailed reviews describing these proteolytic systems exist and, therefore, only a short synopsis is provided to highlight the role that ROS play in stimulating activation of specific proteases [5,7,15]. Briefly, autophagy is a highly regulated lysosomal pathway for the degradation of organelles and select cytosolic proteins [7]. During autophagic protein breakdown, both organelles (e.g., mitochondria) and cytosolic proteins are packaged into vesicles called autophagosomes; following formation, these vesicles fuse with lysosomes and the autophagosome contents are degraded by lysosomal proteases (i.e., cathepsins). In healthy muscle fibers, autophagy is a tightly controlled proteolytic pathway [7]. However, increased production of ROS in cells accelerates autophagic flux via the induction of autophagy, coupled with an increased expression of key autophagy proteins [15,16].

The ubiquitin–proteasome system is comprised of a core proteasome subunit (20S) that provides an enclosed cavity where proteins are degraded. This 20S subunit is coupled with a regulatory complex (19S) connected to each end [17]. Collectively, the 20S subunit, combined with the 19S regulatory complexes, forms the complete ubiquitin–proteasome complex (labeled as the 26S proteasome). This 26S proteasome degrades proteins that have been ubiquitinated by E3 ligases [17]. Notably, oxidized proteins can also be degraded by the 20S proteasome without undergoing ubquitination [18]. Moreover, oxidative stress can promote protein degradation in several other ways. For example, oxidants can stimulate gene expression of key proteins within the ubiquitin proteasome system, including muscle specific E3 ligases [15].

Calpains are calcium-activated proteases that selectively cleave target proteins [19,20]. Calpain activation occurs due to increased cytosolic levels of free calcium and oxidative stress is an established trigger to promote disturbances in cellular calcium homeostasis [21]. Although 15 different calpains exist in humans, the two primary calpains that contribute to skeletal muscle proteolysis are calpain 1 and calpain 2 [19]. Active calpains are reported to cleave >100 proteins including cytoskeletal proteins (e.g., titin, nebulin), kinases, phosphatases, and oxidized contractile proteins (i.e., actin and myosin) [19,22].

Caspase-3 is the fourth major proteolytic system found in muscle fibers. Caspase-3 can be activated via several interrelated signaling processes and, similar to calpain, oxidative stress is a prominent activator of caspase-3 [21]. Caspase-3 can cleave numerous muscle proteins, including actin and myosin complexes [22]. Moreover, oxidation of muscle contractile proteins increases the susceptibility of these proteins to caspase-3 degradation [22].

To summarize, skeletal muscle mass is regulated by the interplay between the rates of protein synthesis and rates of proteolysis. If follows that skeletal muscle atrophy occurs when the rate of proteolysis exceeds the rate of protein synthesis. Although numerous factors participate in the control of muscle protein synthesis and proteolysis, oxidative stress is a common factor that contributes to muscle atrophy by depressing protein synthesis and accelerating proteolysis [15]. While several sites of oxidant production exist in muscle fibers, mitochondria dysfunction often results in increased ROS emission [23,24,25,26,27]. The next section highlights the theory behind the postulate that mitochondrial dysfunction is an essential contributor to muscle wasting.

## 2. Signaling Links between Mitochondrial Dysfunction and Skeletal Muscle Wasting

The earliest suggestion that mitochondrial dysfunction contributes to skeletal muscle wasting was reported in 1964 [28]. This study documented that mitochondrial dysfunction occurs prior to the appearance of muscle atrophy in denervation-induced muscle wasting; however, no direct evidence was provided that mitochondrial dysfunction contributed to muscle atrophy. Nonetheless, since the original postulate that mitochondrial dysfunction contributes to muscle atrophy, numerous studies have documented signaling connections between mitochondrial dysfunction and muscle wasting in a variety of wasting conditions. This work has been summarized in several recent reviews [23,24,25,26,27] and, therefore, only a synopsis is provided here.

### 2.1. Mitochondrial Signaling Leading to Skeletal Muscle Wasting: Premise

**Figure 1:**
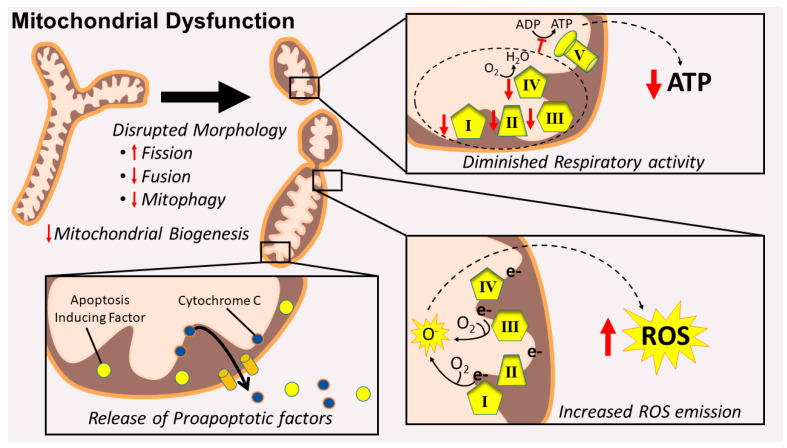
Dysfunctional mitochondria display a disrupted morphology that appears swollen and fragmented compared to healthy mitochondria. These alterations coincide with an impaired respiratory capacity (e.g., decreased mitochondrial complex activity) that results in diminished ATP production, increased mitochondrial ROS emissions, and the release of mitochondria-derived proapoptotic factors.

Mitochondrial dysfunction can contribute to skeletal muscle wasting in at least three ways: (1) increased mitochondrial production of ROS; (2) mitochondrial release of proapoptotic factors; and (3) mitochondrial damage resulting in a reduced production of ATP via oxidative phosphorylation (Figure 1).

The following examines increased mitochondrial ROS production. Numerous preclinical studies provide evidence that increased mitochondrial ROS emission accompanies muscle wasting in several conditions (e.g., disease, prolonged inactivity, etc.). For instance, it is well established that prolonged skeletal muscle inactivity is associated with increased mitochondrial ROS emissions [29,30,31,32]. Direct evidence also indicates that denervation-induced skeletal muscle wasting is also accompanied by increased mitochondrial ROS production [33]. Further, increases in mitochondrial ROS emissions are observed with age-related loss of skeletal muscle mass (i.e., sarcopenia), cancer, and treatment with doxorubicin (a chemotherapeutic drug) [30,34,35]. Together, these studies confirm that increased mitochondrial ROS production accompanies the muscle wasting associated with these conditions.

**Figure 2:**
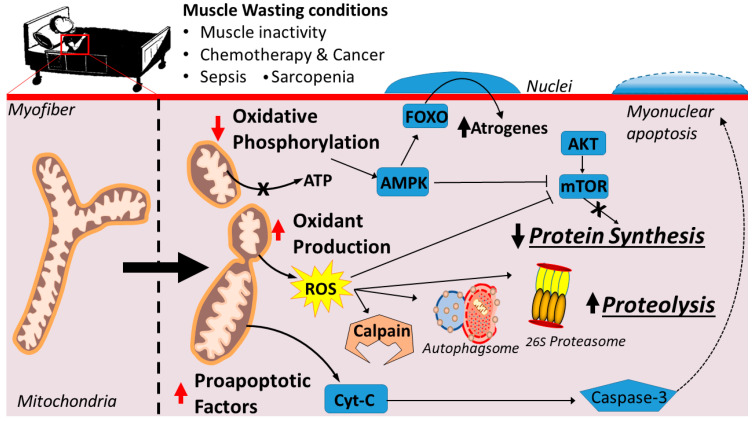
Mitochondrial dysfunction arising from conditions of prolonged muscle inactivity, aging, chemotherapy, cancer, and sepsis, resulting in muscle wasting. Decreased respiratory capacity decreases ATP content and activates AMPK signaling. Increased ROS emissions activate the major proteolytic pathways and inactivates muscle protein synthesis pathways. The release of mitochondrial-derived proapoptotic factors activates caspase-3 and mediates myonuclear apoptosis.

A chronic increase in mitochondrial ROS production can promote muscle wasting by inhibiting muscle protein synthesis and accelerating proteolysis (Figure 2). As mentioned earlier, oxidative stress can activate all four of the major proteolytic systems (reviewed in [15,36]). Specifically, oxidative stress can elevate proteolysis in three independent ways. First, oxidative stress often results in increased cytosolic free calcium, and elevated cytosolic calcium can activate both calpains and caspase-3 [19,20,37,38]. Second, redox disturbances can stimulate several transcriptional activators that promote expression of genes involved in proteolysis (i.e., atrogenes) [39,40]. Finally, oxidative stress can also accelerate proteolysis by oxidizing muscle proteins and increasing their susceptibility to proteolytic breakdown by calpains, caspase-3, and the ubiquitin–proteasome system [18,22].

In addition to accelerating proteolysis, oxidative stress can also contribute to muscle wasting by depressing protein synthesis in skeletal muscle fibers. Indeed, numerous studies conclude that exposure of cells to oxidants depresses protein synthesis [41,42,43]. This oxidative stress-induced depression of protein synthesis is postulated to occur due to depressed mRNA translation because of decreased anabolic signaling through the Akt/mTOR pathway [44]. Recent evidence also links oxidative stress to depressed protein synthesis in skeletal muscles in vivo [12,45]. Collectively, these studies provide evidence that oxidative stress depresses cellular protein synthesis in both in vitro and in vivo.

### 2.2. Mitochondrial Damage Results in the Release of Proapoptotic Factors

Numerous factors, including cytosolic calcium levels and elevated mitochondrial ROS production, can result in permeabilization of the mitochondria outer membrane, resulting in the release of pro-apoptotic factors [46]. For example, permeabilization of the mitochondrial membrane results in the release of cytochrome c, which activates caspase-3 and, thus, leads to the accelerated breakdown of muscle contractile proteins and myonuclear apoptosis [22,46]. Conceptually, the loss of myonuclei within skeletal muscle fibers could diminish protein synthesis by depressing the transcriptional capacity within the fiber [24]. This premise is supported by experiments demonstrating that knockout of caspase-3 in skeletal muscles protects against denervation-induced muscle atrophy by suppressing apoptosis and the loss of myonuclei [44].

### 2.3. Mitochondrial Dysfunction Results in Energy Stress

Many conditions that result in muscle wasting are associated with mitochondrial dysfunction and a compromised ability to produce ATP [25,29,31,47,48]. Dysfunctional mitochondria exhibit an impaired capacity for oxidative phosphorylation (i.e., state 3 respiration), which can result in low ATP levels in the fibers. Importantly, low levels of ATP can depress muscle protein synthesis and accelerate proteolysis [28,47] (Figure 2). In reference to ATP and protein synthesis, energy is required for protein synthesis and, therefore, low energy levels in the muscle fiber could limit the production of new proteins. Moreover, low energy levels in muscle fibers are also associated with increased AMP-kinase (AMPK) activity, which is associated with inhibition of mTORC directly or indirectly (reviewed in [49]). Nonetheless, studies in bacteria reveal that, during periods of low energy levels in the cell, protein synthesis continues at a level that allows the cell to adapt to a lower energy condition [50]. However, it remains unknown if the low energy levels induced by prolonged muscle inactivity are a primary contributor to the depressed protein synthesis that occurs during inactivity-induced muscle atrophy.

Note that low energy levels in muscle fibers can also promote accelerated protein breakdown by influencing the AMPK/FOXO3 axis (Figure 2). Briefly, AMPK is sensitive to cellular energy levels, such that AMPK activity increases in cells during conditions of low ATP availability [25,51]. Active AMPK promotes the activation of the FoxO3 which is a transcriptional activator responsible for increased expression of key atrogenes involved in the ubiquitin proteasome system (e.g., atrogin-1 and muscle ring finger-1) and autophagy (e.g., LC3) [52]. Hence, it is feasible that AMPK-induced activation of FOXO3 accelerates muscle protein breakdown by expression of proteins involved in both the ubiquitin–proteasome system and autophagy [25,53].

To summarize, the mitochondrial dysfunction that occurs during muscle wasting can contribute to fiber atrophy in at least three ways: (1) increased ROS production; (2) release of proapoptotic factors; and (3) decreased oxidative phosphorylation capacity. Notably, it is likely that these three signaling pathways do not operate independently. Indeed, increased mitochondrial production of ROS can contribute to both increased release of proapoptotic factors from the mitochondria and the low energy stress-induced activation of AMPK, resulting in increased expression of select atrogenes [54]. The remaining sections of this review will debate the experimental evidence that indirectly and directly links mitochondrial dysfunction to the muscle wasting that occurs in response to prolonged muscle inactivity, select diseases, and pharmacological agents used in the treatment of cancer.

## 3. Mitochondrial Dysfunction and Skeletal Muscle Atrophy

The previous segments highlighted the cellular signaling networks that provide a mechanistic connection between mitochondrial dysfunction and skeletal muscle wasting. The final segments of this review will examine the evidence connecting mitochondrial dysfunction to skeletal muscle atrophy due to prolonged muscle inactivity, aging, treatment with chemotherapeutic drugs, and specific diseases known to foster muscle wasting. We begin with a discussion of the evidence linking mitochondrial dysfunction to inactivity-induced muscle atrophy.

### Mitochondrial Dysfunction Resulting in Increased Mitochondrial Ros Emission Promotes Inactivity-Induced Muscle Atrophy

Prolonged skeletal muscle inactivity is associated with both muscle atrophy and a reduction in maximal muscle force production. Common clinical conditions resulting in muscle inactivity include prolonged mechanical ventilation, bedrest, and limb immobilization. For example, intensive care unit patients that are provided respiratory support via mechanical ventilation experience inactivity of both inspiratory muscles (i.e., diaphragm) and limb muscles. In contrast, limb immobilization and bed rest result in selective atrophy of the affected locomotor muscles. It is well known that disuse atrophy occurs due to both a decrease in muscle protein synthesis and increased proteolysis [12,16,55,56,57,58,59,60,61,62]. The cell signaling events that prompt inactivity-induced muscle atrophy remain an active area of study, and growing evidence indicates that oxidative stress is an important stimulus that can promote muscle atrophy.

The first evidence that oxidative stress contributes to inactivity-induced muscle wasting appeared over 30 years ago [63]. Since this landmark discovery, numerous studies have since confirmed this observation and, while debate exists as to whether oxidative stress is essential for disuse muscle atrophy to occur, growing evidence demonstrates that oxidants depress muscle protein synthesis and accelerate proteolysis [12,13,21,31,32,40,48,63,64,65,66,67,68]. Importantly, many studies demonstrate that select antioxidants can partially or completely rescue skeletal muscles from inactivity-induced muscle atrophy (reviewed in [15,36]). Together, this evidence solidifies the notion that oxidative stress is an important promoter of disuse muscle atrophy.

The cellular sites for oxidant production in muscle fibers exposed to prolonged inactivity has received widespread investigation; these studies reveal that oxidants are produced from several sources, including NADPH oxidases, xanthine oxidase, and mitochondria [29,31,32,48,69,70]. However, mitochondrial ROS production plays a dominant role in inactivity-induced oxidative stress in muscle fibers [29,69,70]. Indeed, treatment of animals with a mitochondrial-targeted antioxidant prevents the inactivity-induced increase in mitochondrial ROS emission and protects against inactivity-induced depression of muscle protein synthesis and accelerated proteolysis [12,31,32,48].

The mechanisms responsible for increased mitochondrial ROS emission within inactive muscle fibers have been widely debated, and several rival hypotheses exist (see references [23,25,26,71,72,73] for details). A detailed discussion of these mechanisms exceeds the scope of this report, but a short summary is warranted. Briefly, prolonged muscle inactivity has been hypothesized to promote increased mitochondrial dysfunction and increased ROS production in at least five different ways: (1) Energy oversupply, resulting in an abundance of electron donors and increased oxidant production; (2) impaired fission/fusion events, leading to mitochondrial dysfunction and increased ROS production; (3) mitochondrial calcium overload, leading to dysfunction and accelerated oxidant production; (4) JAK/STAT signaling-induced increases in mitochondrial ROS production; and (5) activation of NADPH oxidase 2 (NOX2) in muscle fibers, resulting in a cross-talk between NOX2 and mitochondria whereby activation of NOX2 promotes an increase in mitochondrial ROS production. What follows is a synopsis of each of these proposed mechanisms responsible for skeletal muscle inactivity-induced mitochondrial dysfunction.

The metabolic oversupply hypothesis evolved from the observation that, following prolonged mechanical ventilation in humans, inactive diaphragm muscle fibers exhibit increased intramuscular lipid (i.e., triglycerides) content [74]. Therefore, in inactive muscle fibers, this increase in energetic substrate supply will exceed the metabolic demand, resulting in an accumulation of electrons entering the electron transport chain. The end-result of these events is increased leakage of electrons from the electron transport chain and increased mitochondrial ROS production [75,76,77]. Nonetheless, it remains unclear if this increased lipid content in muscle fibers is directly responsible for mitochondrial dysfunction in the diaphragm.

Disruption of the mitochondrial network is another common hallmark of mitochondrial dysfunction during catabolic conditions, such as inactivity and various other forms of muscle wasting (reviewed in [26,27]). The dynamic control of the mitochondrial network in skeletal muscle fibers is regulated by the balance of mitochondrial biogenesis, fusion, and fission [26,27]. For example, muscle inactivity resulting from denervation is associated with changes in the expression of key fission and fusion proteins that results in the disruption of the mitochondrial network [78]. Similar findings have been reported in numerous other forms of muscle inactivity (reviewed in [25,27,79]). Indeed, evolving evidence suggests that mitochondrial fission and remodeling of mitochondria play a contributory role in skeletal muscle atrophy due to inactivity, aging, and several chronic diseases [25,27,53,79,80].

Prolonged skeletal muscle inactivity is associated with calcium release from the sarcoplasmic reticulum, resulting in increased cytosolic levels of free calcium [81]; this elevation in cytosolic calcium promotes calcium uptake into the mitochondria and resultant mitochondrial depolarization [82]. Mitochondrial calcium overload is often associated with increased mitochondrial ROS production and activation of the mitochondrial permeability transition pore [82,83,84,85]. Therefore, mitochondrial calcium overload is a proposed mechanism for explaining the mitochondrial dysfunction associated with prolonged muscle inactivity (reviewed in [38]).

Activation of the Janus kinase (JAK)/signal transducer and activator of transcription (STAT) pathway has been shown to promote mitochondrial dysfunction, resulting in increased ROS production in skeletal muscle fibers [86,87,88]. For example, both animal and human studies have confirmed that prolonged mechanical ventilation and the ensuing diaphragmatic inactivity results in activation of the JAK-STAT pathway in diaphragm muscle fibers [87,88]. In particular, activation of JAK results in phosphorylation of STAT3; active STAT3 can then translocate into mitochondria and promote increased ROS production via modulation of the electron transport chain [87,88].

Finally, activation of NOX2 in muscle fibers results in increased mitochondrial ROS production [89]. NOX2-induced ROS production results in a cross-talk between NOX2 and mitochondria, whereby NOX2 production of superoxide promotes increased mitochondrial ROS emission [89]. While NOX2 can be activated in skeletal muscles in several ways, signaling through activation of the angiotensin II type I receptor may play an important role in catabolic conditions [90]. Nonetheless, the precise link between active NOX2 and mitochondrial ROS emission remains unclear.

In summary, five different mechanisms have been proposed to explain the link(s) between prolonged skeletal muscle inactivity and increased mitochondrial ROS emission, and it is feasible that several of these mechanisms may work in concert to promote increases in mitochondrial ROS production. Regardless of the mechanism(s) responsible for inactivity-induced mitochondrial dysfunction, convincing evidence exists that mitochondrial dysfunction results in increased mitochondrial ROS emission and is a key player in promoting inactivity-induced muscle wasting.

## 4. Mitochondrial Dysfunction and Sarcopenia

Sarcopenia is defined as the age-related loss of skeletal muscle mass and function [91]. Clinically, patients with sarcopenia are identified as individuals with age-related loss of skeletal muscle mass that is two standard deviations (or more) below the mean of healthy, middle aged individuals [92]. Estimates of the incidence of sarcopenia vary across studies, but a large-scale investigation involving >4650 subjects reported that ~35% of women and ~75% of men over the age of 60 years meet the criteria for sarcopenia [93]. Unfortunately, the incidence of sarcopenia increases above age 70, and ~52% of women and ~88% of men are labeled as sarcopenic at age 80 or higher. This age-related decline in muscle mass has significant consequences, as sarcopenia is a risk factor for the loss of both mobility and independence; moreover, sarcopenia is associated with increased comorbidities that pose a major healthcare challenge for older adults [94].

The mechanisms responsible for sarcopenia are complex and likely involve several factors, including mitochondrial dysfunction, oxidative stress, satellite cell dysfunction, neurological deficiencies (e.g., impaired neuromuscular junctions), chronic low-grade inflammation, and diminished anabolic signaling in muscle [26,95,96,97,98]. Although sarcopenia has a complex etiology, oxidative stress has been suggested to be a key factor contributing to age-related muscle loss (reviewed in [99]). Although ROS can be produced in a variety of subcellular sites (reviewed in [100]), mitochondria isolated from skeletal muscle of senescent animals exhibit increased production of ROS [101,102]. Further, aging is associated with dysfunction of skeletal muscle mitochondria, including impaired oxidative phosphorylation, reduced mitochondrial DNA content, accumulation of mutated mitochondria DNA, dysfunctional fission/fusion, and impaired autophagy (i.e., mitophagy) [103,104,105,106,107]. Together, these observations form the basis for the premise that mitochondrial dysfunction and increased mitochondrial ROS production are responsible for the sarcopenic phenotype [99].

Numerous studies confirm that sarcopenic muscles exhibit impaired mitochondrial fusion and fission (reviewed in [27]). For example, mitochondria from aged skeletal muscles of both rodents and humans display morphological abnormalities that include both mitochondrial enlargement and fragmentation [108,109]. Further, compared to muscles from young adult animals, fibers from senescent skeletal muscles exhibit fusion/fission abnormalities, as evidenced by lower abundance of both mitochondrial fusion proteins (e.g., mitofusins 1 and 2 (Mfn1, Mfn2) and optic atrophy protein 1 (OPA1)), as well as the fission protein dynamin-related protein 1 (DRP1) [110,111,112]. Importantly, an age-related decline in OPA1 has also been reported in humans [112]. This age-related decline in fusion/fission proteins is significant because deletion of Mfn2 or OPA1 results in skeletal muscle atrophy in young animals. Collectively, these results support the concept that an age-related impairment of key fusion and fission proteins is a potential contributor to sarcopenia (reviewed in [25,26]).

As discussed previously, it is widely reported that mitochondrial health in skeletal muscle fibers declines with age. The maintenance of mitochondrial health over the lifespan is dependent upon the removal of damaged mitochondria via mitophagy and mitochondrial biogenesis to replace these damaged mitochondria [26,80]. It follows that a decline in mitophagy in skeletal muscle leads to the accumulation of damaged and dysfunctional mitochondria [27]. In this regard, numerous mitophagy regulators decline with age in sarcopenic muscles of both rodents and humans, and this decrease correlates to walking speed in the frail elderly [104,113,114,115]. Hence, an age-related decline in mitophagy is predicted to contribute to sarcopenia. A potential causal link between depressed mitophagy and muscle atrophy is that dysfunctional mitochondria produce higher levels of ROS [34,101,102]. This is important because oxidative stress has been proposed to be a key contributor to sarcopenia [99]. Evidence that mitochondrial dysfunction contributes to muscle dysfunction in aged animals comes from two independent studies revealing that treatment with the mitochondrial targeted antioxidant peptide SS-31 protects against age-related decline of muscle endurance in senescent animals [116,117]. In contrast to these results, a recent study concludes that pharmacological attenuation of age-related increases in mitochondrial ROS emission (i.e., treatment with SS31) does not rescue age-related muscle atrophy but does protect against oxidative damage and a decline in mitophagy in aged muscles [34]. However, it is important to note that the animals treated with this pharmacological intervention were treated for only four months, beginning late in the animals’ lives. Future studies are required to determine whether lifelong treatment with this mitochondrial-targeted peptide would have protected against sarcopenia.

To summarize, the etiology of sarcopenia is complex and likely involves the interaction of a variety of factors, including a decline in mitochondrial dysfunction. Indeed, accumulating evidence suggests that an age-related increase in mitochondrial dysfunction is a key contributor to sarcopenia.

## 5. Role of Mitochondrial Dysfunction in Chemotherapy-Induced Muscle Wasting

Doxorubicin (DOX) is an anthracycline antibiotic that is widely used as an antitumor agent in the treatment of human cancers. While DOX is highly effective in the treatment of numerous cancers (e.g., lymphoma, leukemia, breast, and Kaposi’s sarcoma), DOX is cytotoxic and promotes the rapid wasting of both cardiac and skeletal muscle fibers. Indeed, a common clinical risk of using this highly effective anticancer drug is the development of cardiomyopathy [118]. Treatment with DOX also promotes rapid atrophy in all skeletal muscle fiber types, with no preference between fiber types in the rate of atrophy [30].

The mechanism(s) responsible for DOX-induced cardiac and skeletal muscle wasting has been extensively studied and evidence reveals that DOX-induced toxicity to both cardiac and skeletal muscle fibers is driven by increased mitochondrial ROS production and resultant oxidative stress (reviewed in [119,120]). Expressly, although DOX can promote ROS production in cells via several pathways, mitochondrial ROS production is the primary source of DOX-induced ROS production in both cardiac and skeletal muscle fibers [30]. DOX-induced increase in mitochondrial ROS production is propelled by DOX accumulation in mitochondria, resulting in DOX redox cycles on complex I resulting in the subsequent production of superoxide radicals [121,122]. This DOX-induced rise in mitochondrial ROS emission promotes the activation of all four major proteolytic systems and a rapid rise in muscle protein degradation [16,30,123,124,125]. Although DOX administration activates all proteolytic systems in skeletal muscle, active calpain plays a particularly important role in the loss of skeletal muscle protein [30].

Support for the idea that increased mitochondrial ROS production is essential for DOX-induced atrophy of both cardiac and skeletal muscle fibers comes from several lines of evidence. For example, treatment of C2C12 myotubes with SS-31, a mitochondrial-targeted protective peptide, prevents DOX-induced myotube atrophy [123]. Similarly, preclinical studies confirm that treatment of rodents with SS-31 prevents DOX-induced activation of cellular proteases and atrophy of both cardiac and skeletal muscle fibers [30,126]. Similarly, treatment of rodents with MitoQ, a mitochondrial-targeted antioxidant, protects against DOX-induced cardiac dysfunction [127].

To summarize, although DOX is a highly effective chemotherapeutic agent against numerous solid tumors, treatment of cancer patients with DOX is limited by the drugs’ toxic effects on both cardiac and skeletal muscle. In this regard, compelling evidence reveals that treatment with DOX results in both mitochondrial dysfunction and increases in mitochondrial ROS emission within both cardiac and skeletal muscle fibers. Importantly, a growing number of investigations indicate that increases in mitochondrial production of ROS is a required trigger to promote DOX-induced muscle wasting.

## 6. Mitochondria and Cancer Cachexia

Cancer cachexia is characterized by the loss of skeletal muscle mass with or without the loss of fat mass. Clinically, cancer cachexia is defined as an involuntary loss of >5% of total body weight within 6 months or a body mass index (BMI) of <20 [128]. It is estimated that cancer cachexia affects ~30% of all cancer patients, with prevalence ranging from ~11–89% depending on the type of cancer [129,130,131]. The prevalence and degree of muscle wasting that occurs with cancer cachexia varies and is dependent upon cancer type and disease progression. Body weight loss can range from a low weight loss (<5% of total body weight, termed prechachexia) to severe with body weight loss exceeding ~18% of total body mass in a 6 month period [132]. Cancer cachexia is commonly observed in gastrointestinal cancer (e.g., pancreatic cancer) as well as lung and prostate cancer. Importantly, cancer cachexia is also associated with depressed appetite; nonetheless, conventional nutritional support does not compensate cancer-mediated muscle wasting [128]. Unfortunately, cancer-mediated muscle wasting is associated with higher mortality rates in cancer patients [133].

While significant progress has been made in our understanding of cancer cachexia in recent years, the complexities of various cancer pathologies and patient populations has mired the development of treatment options. Over 100 types of cancer have been identified with varying pathologies. This point is further complicated by the fact that similar cancer types can also manifest diverging signaling pathways. For instance, a recent report by Nosacka et al. demonstrated that cancerous xenografts from four different patients diagnosed with pancreatic ductal adenocarcinoma resulted in distinct physiological responses when transplanted in rodents [134]. Moreover, studies of human cancer patients are difficult to interpret because of age-related frailty of the patient population and pharmacological treatments that confound results (e.g., doxorubicin). Nonetheless, accruing evidence reveals several commonalities with cancer cachexia.

Findings from both preclinical and clinical studies confirm that the hallmarks of cancer cachexia are decreased myofiber cross-sectional area, disrupted muscle ultrastructure, and increased fibrosis [35,135,136,137,138]. Both depressed protein synthesis and accelerated proteolysis have been observed with muscle wasting due to cancer cachexia [139,140,141,142]. While the factors that contribute to cancer-induced muscle wasting are believed to be multifactorial, recent evidence has implicated mitochondrial dysfunction as a key contributor to cancer cachexia.

Reports from both preclinical and human studies reveal that mitochondrial morphology is disrupted with cancer cachexia, displaying a swollen phenotype [136,143,144,145]. Observations of disrupted mitochondrial morphology are supported by reports demonstrating alterations in mitochondrial fusion and fission machinery with several reports showing increased expression of Fis1 and decreased Parkin [145,146,147]. Additionally, decreased mitochondrial respiration and mitochondrial complex activity have also been observed with cancer cachexia [138,142,143,148,149].

With regard to the evidence that mitochondrial dysfunction contributes to cancer cachexia, several reports have provided compelling arguments for the involvement of mitochondrial dysfunction with cancer cachexia. Recently, Brown et al. provided evidence that mitochondrial dysfunction precedes the development of cachexia in rodent models of lung and colorectal cancer [138]. Further, pharmacological targeting of mitochondria with the small peptide SS-31 has been shown to attenuate muscle wasting in a rodent model of colon cancer [35]. Collectively, these studies implicate mitochondrial dysfunction as a key contributor to muscle wasting. In theory, mitochondrial dysfunction can contribute to cancer cachexia through chronic elevation in mitochondrial ROS emission and disrupted ATP producing capacity.

As discussed previously, chronic elevation in mitochondrial ROS emissions can elicit muscle wasting. Evidence from preclinical models show that mitochondrial ROS emissions increase with cancer cachexia and are accompanied by increased markers of oxidative stress [35,138,139,150,151]. In contrast to these findings, some studies have reported decreased markers of muscle oxidative stress, and that exacerbated oxidative stress does not accentuate cancer cachexia [146,152]. Perhaps one explanation for this discrepancy between experimental findings can be explained by evidence of the time course of mitochondrial dysfunction. For instance, it appears that mitochondrial ROS emissions are elevated within the first three weeks of cancer induction before returning to baseline in rodent models of cancer cachexia [138,139]. Hence, it is plausible that muscle atrophy is first initiated by early elevations in mitochondrial ROS emissions. In support of this, evidence in cell culture demonstrates that incubation of cells with media derived from kidney cancer cells increases mitochondrial ROS production and induces myotube atrophic response [151].

A depressed ability for mitochondria to produce ATP may also contribute to cancer cachexia. In this regard, several studies have shown diminished mitochondrial complex activity and mitochondrial respiration [138,142,143,148,149,153]. Indeed, ATP content is decreased in muscle undergoing cancer-induced cachexia [149]. As discussed previously, low levels of ATP can diminish energy availability for processes of protein synthesis, as well as leading to increased AMPK activation. Notably, despite evidence of cachexia-induced decreases in mitochondrial respiration, increasing mitochondrial volume via overexpression of PGC-1α was unable to rescue cancer-induced muscle wasting [154]. Further, AMPK activity does not appear to increase until later in the progression of cancer cachexia, suggesting that AMPK activation occurs after the onset of muscle atrophy [155]. Nonetheless, disrupted energy producing capacity may play a role in the cancer-induced muscle wasting.

While compelling evidence exists supporting the tenet that mitochondrial dysfunction contributes to various cancer types, the highly diverging physiological response between different cancer types warrants consideration and limits our current understanding. Evidence from preclinical models that mitochondrial dysfunction precedes cancer cachexia, and that mitochondrial targeted pharmacological agents attenuate cancer cachexia, supports this notion. Nonetheless, many cancer types exist with varying pathophysiology. Future research is required to delineate the precise role that mitochondria play in cancer cachexia.

## 7. Evidence Linking Mitochondrial Dysfunction with Sepsis-Induced Muscle Wasting

Sepsis is a pathological condition characterized by a systemic inflammatory response due to infection of microbial origin. Sepsis is a life threatening condition that can result in organ failure of one or more organ systems, and is estimated to affect more than 48 million patients worldwide [156]. Among the threat of damage to organ systems, sepsis evokes skeletal muscle wasting of both respiratory and locomotor muscles [157,158,159,160]. This is problematic, as this muscle wasting likely contributes to impaired mobility and reduced quality of life in sepsis survivors [161].

While the factors that contribute to sepsis-induced muscle wasting are likely multifactorial, mitochondrial dysfunction has emerged as key contributor to this muscle wasting. The first evidence that mitochondrial dysfunction plays a major role in muscle wasting was reported by Brealey et al. [162]. In this seminal report, the severity of sepsis was found to be associated with mitochondrial dysfunction in skeletal muscle; mitochondria in sepsis patients exhibited diminished mitochondrial complex activity, depressed antioxidant capacity, and lower ATP concentrations. Hence, bioenergetics failure was implicated to contribute to the severity of sepsis-induced muscle wasting [162]. Indeed, numerous reports have demonstrated the extensive mitochondrial dysfunction that occurs with sepsis-induced muscle wasting [163,164,165,166,167,168,169,170]. Mitochondrial dysfunction has been postulated to contribute to sepsis-induced muscle wasting in three ways: (1) diminished energy producing capacity; (2) increased oxidative stress; and (3) deficient satellite cell function.

Sepsis has been well documented to result in a decreased capacity for mitochondria to produce ATP through oxidative phosphorylation in skeletal muscle. Preclinical and human studies have consistently shown that mitochondrial complex gene expression, protein abundance, and activity are decreased with sepsis [162,163,165,167,171,172]. This decrease in oxidative phosphorylation (OXPHOS) machinery also corresponds to decreased ATP content in muscle [162,165,171,173,174,175]. In addition to the decrease in OXPHOS machinery, decreased energy availability may also be exacerbated by a decreased ability to distribute energy from the mitochondria throughout the myofiber. For instance, mitochondrial creatine kinase activity and protein abundance are decreased with sepsis, which would limit the ability to transport ATP out of the mitochondria into the cytosol [176]. Collectively, the inability of dysfunctional mitochondria to provide energy to the cell may result in the muscle atrophy, as discussed previously.

While the cause(s) driving diminished mitochondrial respiratory capacity has not been fully elucidated, an increased production of free radicals has been purported to play an inhibitory role in mitochondrial respiration during sepsis [177]. Notably, cross-talk can occur between sources of oxidant production (e.g., NADPH oxidase) and mitochondria that result in increased mitochondrial ROS emissions; oxidation of mitochondria increases mitochondrial-derived ROS production, resulting in a viscous cycle of oxidative stress that results in increased muscle proteolysis [36]. Sepsis is reported to increase oxidant emission from ROS generating enzymes, such as NADPH oxidase and nitric oxide synthase [177,178]. In this regard, inhibition of nitric oxide synthase and administration of free radical scavengers has been shown to prevent sepsis-induced mitochondrial dysfunction [177]. Further, nitric oxide is also capable of independently inhibiting complex I activity in mitochondria [179]; nitric oxide modification of mitochondria can result in decreased mitochondrial respiration. Indeed, knockout mice that lack the inducible nitric oxide synthase (iNOS) isoform are protected from mitochondrial dysfunction in response to sepsis [180]. Hence, activation of ROS generating enzymes may play a role in the initial development of mitochondrial dysfunction during sepsis.

While other sources of ROS may play a role in triggering mitochondrial dysfunction during sepsis, mitochondria themselves are major contributors to ROS production in myofibers. Indeed, increased mitochondrial ROS emissions may contribute to sepsis-induced muscle wasting. Several reports have shown that muscle mitochondrial ROS emissions are increased with sepsis [172,175,181,182]. The importance of sepsis-induced mitochondrial ROS is demonstrated by time-course studies showing that increases in mitochondrial superoxide production correlate with the decreases in muscle force production during sepsis [172]. Moreover, mitochondrial targeted antioxidants prevent sepsis-induced contractile dysfunction in diaphragm muscle [175,182]. These studies provide strong evidence for the importance of mitochondrial dysfunction with sepsis; however, these studies did not measure muscle cross-sectional area, and direct evidence on mitochondrial dysfunction in sepsis-induced muscle wasting is limited.

In regard to the direct evidence implicating mitochondrial dysfunction as a critical mediator of sepsis-induced muscle wasting, a recent report reveals that overexpression of parkin, a protein responsible for mitophagy (i.e., mitochondrial autophagy), protects muscle against sepsis-induced wasting [168]. Mitochondria are observed to present a swollen appearance and disorganized morphology in muscle during sepsis [165,168,170,176,183]. However, parkin overexpression during sepsis attenuated altered mitochondrial morphology and prevented myofiber atrophy [168]. The protective effects of parkin overexpression likely occur through increased removal of dysfunctional mitochondria via mitophagy; however, parkin overexpression was also noted to increase Nrf2, a key transcriptional regulator of antioxidant enzymes, which may have contributed to the protective effects. Future studies are required to further asses the protective role of mitophagy during sepsis.

Lastly, it should also be noted that mitochondrial dysfunction may contribute to sepsis-induced myopathy by affecting muscle satellite cells. A recent report reveals that mitochondria in satellite cells become dysfunctional and that this dysfunction persists following recovery from sepsis [173]. Skeletal muscle is observed to have a blunted regenerative capacity following sepsis, and survivors can exhibit muscle weakness five years following recovery from sepsis [173,184]. In this regard, mitochondrial dysfunction in muscle stem cells is attributed to play a role in the blunted regenerative capacity of muscle following sepsis. The authors demonstrate that engrafting mescenchymal stem cells is capable of improving mitochondrial function, and restores the regenerative capacity of skeletal muscle [173]. The role of mitochondrial dysfunction in satellite cells further highlights the multifactorial role of mitochondria in sepsis-induced myopathy.

## 8. Summary and Future Directions

The prediction that mitochondrial dysfunction is a primary factor contributing to skeletal muscle atrophy originated in the mid-1900s. Nonetheless, specific evidence demonstrating that mitochondrial damage/dysfunction contributes to numerous forms of muscle wasting was not available until the early 2000s. Specifically, direct evidence connecting mitochondrial dysfunction to muscle wasting due to disease, aging, chemotherapy, and disuse muscle atrophy has steadily emerged over the past decade. Indeed, compelling support now exists that mitochondrial dysfunction contributes to muscle wasting in a variety of diseases (cancer and sepsis), aging, cancer chemotherapy, and muscle atrophy due to prolonged periods of muscle inactivity.

While numerous reports link mitochondrial damage/dysfunction to muscle wasting, many questions remain unanswered. For example, limited information exists about the mechanisms responsible for the skeletal muscle mitochondrial dysfunction that occurs due to disease, aging, and during prolonged inactivity. Further, it remains unclear as to whether both subsarcolemmal and intermyofibrillar mitochondria become dysfunctional during conditions that promote muscle wasting.

Additional research is also needed to identify specific therapeutic interventions that can protect against mitochondrial dysfunction and prevent muscle atrophy. For example, although a few studies suggest that specific mitochondrial-targeted peptides (i.e., SS-31) can protect against muscle wasting during prolonged inactivity and in response to treatment with doxorubicin, additional studies are required to determine whether mitochondrial-targeted treatments are effective in preventing muscle wasting during long durations of muscle inactivity (weeks to months) and during prolonged treatment with chemotherapeutic drugs (e.g., doxorubicin). Moreover, more experiments are needed to establish whether mitochondrial-directed compounds can prevent sepsis-induced muscle wasting. Similarly, while experiments indicate that treatment with mitochondrial-targeted peptides during late senescence do not prevent age-related muscle atrophy, it remains unclear whether an appropriate intervention can protect against sarcopenia if treatment begins early in life.

Another important topic for future investigations relates to the observation that cancer-induced increases in skeletal muscle mitochondrial ROS emissions returns to baseline over time. This finding raises two key questions. First, what are the mechanism(s) responsible for this time-dependent fluctuation in cancer-induced increase mitochondrial ROS emission? Second, what is the significance of this fluctuation in mitochondrial ROS production in promoting sepsis-induced muscle atrophy?

Finally, it remains unknown whether increased mitochondrial ROS production plays a key role in the regulation of fission and fusion in skeletal muscle mitochondria. Studies that address this and other unanswered questions are required to identify new treatments to prevent muscle wasting due to disease, doxorubicin, aging, and prolonged muscle disuse.

## References

[1] Febbraio M.A., Hiscock N., Sacchetti M., Fischer C.P., Pedersen B.K. (2004). Interleukin-6 is a novel factor mediating glucose homeostasis during skeletal muscle contraction. Diabetes.

[2] Meyer C., Dostou J.M., Welle S.L., Gerich J.E. (2002). Role of human liver, kidney, and skeletal muscle in postprandial glucose homeostasis. Am. J. Physiol. Endocrinol. Metab..

[3] Srikanthan P., Karlamangla A.S. (2014). Muscle mass index as a predictor of longevity in older adults. Am. J. Med..

[4] Weijs P.J., Looijaard W.G., Dekker I.M., Stapel S.N., Girbes A.R., Oudemans-van Straaten H.M., Beishuizen A. (2014). Low skeletal muscle area is a risk factor for mortality in mechanically ventilated critically ill patients. Crit. Care.

[5] Bodine S.C., Edward F. (2020). Adolph Distinguished Lecture. Skeletal muscle atrophy: Multiple pathways leading to a common outcome. J. Appl. Physiol..

[6] Sartori R., Romanello V., Sandri M. (2021). Mechanisms of muscle atrophy and hypertrophy: Implications in health and disease. Nat. Commun..

[7] Vainshtein A., Sandri M. (2020). Signaling Pathways That Control Muscle Mass. Int. J. Mol. Sci..

[8] Dupont-Versteegden E.E., McCarthy J.J. (2019). Translational control of muscle mass. J. Appl. Physiol..

[9] Greenbaum D., Colangelo C., Williams K., Gerstein M. (2003). Comparing protein abundance and mRNA expression levels on a genomic scale. Genome Biol..

[10] Tian Q., Stepaniants S.B., Mao M., Weng L., Feetham M.C., Doyle M.J., Yi E.C., Dai H., Thorsson V., Eng J. (2004). Integrated genomic and proteomic analyses of gene expression in Mammalian cells. Mol. Cell. Proteomics.

[11] Ogasawara R., Jensen T.E., Goodman C.A., Hornberger T.A. (2019). Resistance Exercise-Induced Hypertrophy: A Potential Role for Rapamycin-Insensitive mTOR. Exerc. Sport Sci. Rev..

[12] Hudson M.B., Smuder A.J., Nelson W.B., Wiggs M.P., Shimkus K.L., Fluckey J.D., Szeto H.H., Powers S.K. (2015). Partial Support Ventilation and Mitochondrial-Targeted Antioxidants Protect against Ventilator-Induced Decreases in Diaphragm Muscle Protein Synthesis. PLoS ONE.

[13] O’Loghlen A., Perez-Morgado M.I., Salinas M., Martin M.E. (2006). N-acetyl-cysteine abolishes hydrogen peroxide-induced modification of eukaryotic initiation factor 4F activity via distinct signalling pathways. Cell Signal..

[14] Zhang L., Kimball S.R., Jefferson L.S., Shenberger J.S. (2009). Hydrogen peroxide impairs insulin-stimulated assembly of mTORC1. Free Radic. Biol. Med..

[15] Powers S.K., Morton A.B., Ahn B., Smuder A.J. (2016). Redox control of skeletal muscle atrophy. Free Radic. Biol. Med..

[16] Smuder A.J., Sollanek K.J., Nelson W.B., Min K., Talbert E.E., Kavazis A.N., Hudson M.B., Sandri M., Szeto H.H., Powers S.K. (2018). Crosstalk between autophagy and oxidative stress regulates proteolysis in the diaphragm during mechanical ventilation. Free Radic. Biol. Med..

[17] Powell S.R. (2006). The ubiquitin-proteasome system in cardiac physiology and pathology. Am. J. Physiol. Heart Circ. Physiol..

[18] Grune T., Reinheckel T., Davies K.J. (1997). Degradation of oxidized proteins in mammalian cells. FASEB J..

[19] Goll D.E., Thompson V.F., Li H., Wei W., Cong J. (2003). The calpain system. Physiol. Rev..

[20] Hyatt H.W., Ozdemir M., Yoshihara T., Nguyen B.L., Deminice R., Powers S.K. (2021). Calpains play an essential role in mechanical ventilation-induced diaphragmatic weakness and mitochondrial dysfunction. Redox Biol..

[21] Whidden M.A., Smuder A.J., Wu M., Hudson M.B., Nelson W.B., Powers S.K. (2010). Oxidative stress is required for mechanical ventilation-induced protease activation in the diaphragm. J. Appl. Physiol..

[22] Smuder A.J., Kavazis A.N., Hudson M.B., Nelson W.B., Powers S.K. (2010). Oxidation enhances myofibrillar protein degradation via calpain and caspase-3. Free Radic. Biol. Med..

[23] Hyatt H., Deminice R., Yoshihara T., Powers S.K. (2019). Mitochondrial dysfunction induces muscle atrophy during prolonged inactivity: A review of the causes and effects. Arch. Biochem. Biophys..

[24] Powers S.K., Wiggs M.P., Duarte J.A., Zergeroglu A.M., Demirel H.A. (2012). Mitochondrial signaling contributes to disuse muscle atrophy. Am. J. Physiol. Endocrinol. Metab..

[25] Romanello V., Guadagnin E., Gomes L., Roder I., Sandri C., Petersen Y., Milan G., Masiero E., Del Piccolo P., Foretz M. (2010). Mitochondrial fission and remodelling contributes to muscle atrophy. EMBO J..

[26] Romanello V., Sandri M. (2015). Mitochondrial Quality Control and Muscle Mass Maintenance. Front. Physiol..

[27] Romanello V. (2020). The Interplay between Mitochondrial Morphology and Myomitokines in Aging Sarcopenia. Int. J. Mol. Sci..

[28] Carafoli E., Margreth A., Buffa P. (1964). Early Biochemical Changes in Mitochondria from Denervated Muscle and Their Relation to the Onset of Atrophy. Exp. Mol. Pathol..

[29] Kavazis A.N., Talbert E.E., Smuder A.J., Hudson M.B., Nelson W.B., Powers S.K. (2009). Mechanical ventilation induces diaphragmatic mitochondrial dysfunction and increased oxidant production. Free Radic. Biol. Med..

[30] Min K., Kwon O.S., Smuder A.J., Wiggs M.P., Sollanek K.J., Christou D.D., Yoo J.K., Hwang M.H., Szeto H.H., Kavazis A.N. (2015). Increased mitochondrial emission of reactive oxygen species and calpain activation are required for doxorubicin-induced cardiac and skeletal muscle myopathy. J. Physiol..

[31] Powers S.K., Hudson M.B., Nelson W.B., Talbert E.E., Min K., Szeto H.H., Kavazis A.N., Smuder A.J. (2011). Mitochondria-targeted antioxidants protect against mechanical ventilation-induced diaphragm weakness. Crit. Care Med..

[32] Talbert E.E., Smuder A.J., Min K., Kwon O.S., Szeto H.H., Powers S.K. (2013). Immobilization-induced activation of key proteolytic systems in skeletal muscles is prevented by a mitochondria-targeted antioxidant. J. Appl. Physiol..

[33] Muller F.L., Song W., Jang Y.C., Liu Y., Sabia M., Richardson A., Van Remmen H. (2007). Denervation-induced skeletal muscle atrophy is associated with increased mitochondrial ROS production. Am. J. Physiol. Regul. Integr. Comp. Physiol..

[34] Sakellariou G.K., Pearson T., Lightfoot A.P., Nye G.A., Wells N., Giakoumaki I.I., Vasilaki A., Griffiths R.D., Jackson M.J., McArdle A. (2016). Mitochondrial ROS regulate oxidative damage and mitophagy but not age-related muscle fiber atrophy. Sci. Rep..

[35] Smuder A.J., Roberts B.M., Wiggs M.P., Kwon O.S., Yoo J.K., Christou D.D., Fuller D.D., Szeto H.H., Judge A.R. (2020). Pharmacological targeting of mitochondrial function and reactive oxygen species production prevents colon 26 cancer-induced cardiorespiratory muscle weakness. Oncotarget.

[36] Powers S.K., Ozdemir M., Hyatt H. (2020). Redox Control of Proteolysis During Inactivity-Induced Skeletal Muscle Atrophy. Antioxid. Redox. Signal..

[37] Gonzalez D., Espino J., Bejarano I., Lopez J.J., Rodriguez A.B., Pariente J.A. (2010). Caspase-3 and -9 are activated in human myeloid HL-60 cells by calcium signal. Mol. Cell. Biochem..

[38] Hyatt H.W., Powers S.K. (2020). Disturbances in Calcium Homeostasis Promotes Skeletal Muscle Atrophy: Lessons From Ventilator-Induced Diaphragm Wasting. Front. Physiol..

[39] Aucello M., Dobrowolny G., Musaro A. (2009). Localized accumulation of oxidative stress causes muscle atrophy through activation of an autophagic pathway. Autophagy.

[40] Li Y.P., Chen Y., Li A.S., Reid M.B. (2003). Hydrogen peroxide stimulates ubiquitin-conjugating activity and expression of genes for specific E2 and E3 proteins in skeletal muscle myotubes. Am. J. Physiol. Cell Physiol..

[41] Alirezaei M., Marin P., Nairn A.C., Glowinski J., Premont J. (2001). Inhibition of protein synthesis in cortical neurons during exposure to hydrogen peroxide. J. Neurochem..

[42] Pham F.H., Sugden P.H., Clerk A. (2000). Regulation of protein kinase B and 4E-BP1 by oxidative stress in cardiac myocytes. Circ. Res..

[43] Shenton D., Smirnova J.B., Selley J.N., Carroll K., Hubbard S.J., Pavitt G.D., Ashe M.P., Grant C.M. (2006). Global translational responses to oxidative stress impact upon multiple levels of protein synthesis. J. Biol. Chem..

[44] Powers S.K., Smuder A.J., Criswell D.S. (2011). Mechanistic links between oxidative stress and disuse muscle atrophy. Antioxid. Redox. Signal..

[45] Marzani B., Balage M., Venien A., Astruc T., Papet I., Dardevet D., Mosoni L. (2008). Antioxidant supplementation restores defective leucine stimulation of protein synthesis in skeletal muscle from old rats. J. Nutr..

[46] Bloemberg D., Quadrilatero J. (2019). Autophagy, apoptosis, and mitochondria: Molecular integration and physiological relevance in skeletal muscle. Am. J. Physiol. Cell Physiol..

[47] Max S.R. (1972). Disuse atrophy of skeletal muscle: Loss of functional activity of mitochondria. Biochem. Biophys. Res. Commun..

[48] Min K., Smuder A.J., Kwon O.S., Kavazis A.N., Szeto H.H., Powers S.K. (2011). Mitochondrial-targeted antioxidants protect skeletal muscle against immobilization-induced muscle atrophy. J. Appl. Physiol..

[49] Thomson D.M. (2018). The Role of AMPK in the Regulation of Skeletal Muscle Size, Hypertrophy, and Regeneration. Int. J. Mol. Sci..

[50] Jewett M.C., Miller M.L., Chen Y., Swartz J.R. (2009). Continued protein synthesis at low [ATP] and [GTP] enables cell adaptation during energy limitation. J. Bacteriol..

[51] Carling D., Zammit V.A., Hardie D.G. (1987). A common bicyclic protein kinase cascade inactivates the regulatory enzymes of fatty acid and cholesterol biosynthesis. FEBS Lett..

[52] Greer E.L., Oskoui P.R., Banko M.R., Maniar J.M., Gygi M.P., Gygi S.P., Brunet A. (2007). The energy sensor AMP-activated protein kinase directly regulates the mammalian FOXO3 transcription factor. J. Biol. Chem..

[53] Romanello V., Sandri M. (2010). Mitochondrial biogenesis and fragmentation as regulators of muscle protein degradation. Curr. Hypertens. Rep..

[54] Kowaltowski A.J., Vercesi A.E. (1999). Mitochondrial damage induced by conditions of oxidative stress. Free Radic. Biol. Med..

[55] Levine S., Nguyen T., Taylor N., Friscia M.E., Budak M.T., Rothenberg P., Zhu J., Sachdeva R., Sonnad S., Kaiser L.R. (2008). Rapid disuse atrophy of diaphragm fibers in mechanically ventilated humans. N. Engl. J. Med..

[56] Nelson W.B., Smuder A.J., Hudson M.B., Talbert E.E., Powers S.K. (2012). Cross-talk between the calpain and caspase-3 proteolytic systems in the diaphragm during prolonged mechanical ventilation. Crit. Care Med..

[57] Paddon-Jones D., Sheffield-Moore M., Cree M.G., Hewlings S.J., Aarsland A., Wolfe R.R., Ferrando A.A. (2006). Atrophy and impaired muscle protein synthesis during prolonged inactivity and stress. J. Clin. Endocrinol. Metab..

[58] Phillips S.M., Glover E.I., Rennie M.J. (2009). Alterations of protein turnover underlying disuse atrophy in human skeletal muscle. J. Appl. Physiol..

[59] Shanely R.A., Van Gammeren D., Deruisseau K.C., Zergeroglu A.M., McKenzie M.J., Yarasheski K.E., Powers S.K. (2004). Mechanical ventilation depresses protein synthesis in the rat diaphragm. Am. J. Respir. Crit. Care Med..

[60] Thomason D.B., Biggs R.B., Booth F.W. (1989). Protein metabolism and beta-myosin heavy-chain mRNA in unweighted soleus muscle. Am. J. Physiol..

[61] Thomason D.B., Booth F.W. (1990). Atrophy of the soleus muscle by hindlimb unweighting. J. Appl. Physiol..

[62] Thomason D.B., Morrison P.R., Oganov V., Ilyina-Kakueva E., Booth F.W., Baldwin K.M. (1992). Altered actin and myosin expression in muscle during exposure to microgravity. J. Appl. Physiol..

[63] Kondo H., Miura M., Itokawa Y. (1991). Oxidative stress in skeletal muscle atrophied by immobilization. Acta Physiol. Scand..

[64] Agten A., Maes K., Smuder A., Powers S.K., Decramer M., Gayan-Ramirez G. (2011). N-Acetylcysteine protects the rat diaphragm from the decreased contractility associated with controlled mechanical ventilation. Crit. Care Med..

[65] Appell H.J., Duarte J.A., Soares J.M. (1997). Supplementation of vitamin E may attenuate skeletal muscle immobilization atrophy. Int. J. Sports Med..

[66] Betters J.L., Criswell D.S., Shanely R.A., Van Gammeren D., Falk D., Deruisseau K.C., Deering M., Yimlamai T., Powers S.K. (2004). Trolox attenuates mechanical ventilation-induced diaphragmatic dysfunction and proteolysis. Am. J. Respir. Crit. Care Med..

[67] Laitano O., Ahn B., Patel N., Coblentz P.D., Smuder A.J., Yoo J.K., Christou D.D., Adhihetty P.J., Ferreira L.F. (2016). Pharmacological targeting of mitochondrial reactive oxygen species counteracts diaphragm weakness in chronic heart failure. J. Appl. Physiol..

[68] McClung J.M., Whidden M.A., Kavazis A.N., Falk D.J., Deruisseau K.C., Powers S.K. (2008). Redox regulation of diaphragm proteolysis during mechanical ventilation. Am. J. Physiol. Regul. Integr. Comp. Physiol..

[69] McClung J.M., Van Gammeren D., Whidden M.A., Falk D.J., Kavazis A.N., Hudson M.B., Gayan-Ramirez G., Decramer M., DeRuisseau K.C., Powers S.K. (2009). Apocynin attenuates diaphragm oxidative stress and protease activation during prolonged mechanical ventilation. Crit. Care Med..

[70] Whidden M.A., McClung J.M., Falk D.J., Hudson M.B., Smuder A.J., Nelson W.B., Powers S.K. (2009). Xanthine oxidase contributes to mechanical ventilation-induced diaphragmatic oxidative stress and contractile dysfunction. J. Appl. Physiol..

[71] Abrigo J., Simon F., Cabrera D., Vilos C., Cabello-Verrugio C. (2019). Mitochondrial Dysfunction in Skeletal Muscle Pathologies. Curr. Protein Pept. Sci..

[72] Romanello V., Scalabrin M., Albiero M., Blaauw B., Scorrano L., Sandri M. (2019). Inhibition of the Fission Machinery Mitigates OPA1 Impairment in Adult Skeletal Muscles. Cells.

[73] Supinski G.S., Schroder E.A., Callahan L.A. (2020). Mitochondria and Critical Illness. Chest.

[74] Picard M., Jung B., Liang F., Azuelos I., Hussain S., Goldberg P., Godin R., Danialou G., Chaturvedi R., Rygiel K. (2012). Mitochondrial dysfunction and lipid accumulation in the human diaphragm during mechanical ventilation. Am. J. Respir. Crit. Care Med..

[75] Anderson E.J., Lustig M.E., Boyle K.E., Woodlief T.L., Kane D.A., Lin C.T., Price J.W., Kang L., Rabinovitch P.S., Szeto H.H. (2009). Mitochondrial H2O2 emission and cellular redox state link excess fat intake to insulin resistance in both rodents and humans. J. Clin. Investig..

[76] Anderson E.J., Yamazaki H., Neufer P.D. (2007). Induction of endogenous uncoupling protein 3 suppresses mitochondrial oxidant emission during fatty acid-supported respiration. J. Biol. Chem..

[77] St-Pierre J., Buckingham J.A., Roebuck S.J., Brand M.D. (2002). Topology of superoxide production from different sites in the mitochondrial electron transport chain. J. Biol. Chem..

[78] Graham Z.A., Harlow L., Bauman W.A., Cardozo C.P. (2018). Alterations in mitochondrial fission, fusion, and mitophagic protein expression in the gastrocnemius of mice after a sciatic nerve transection. Muscle Nerve.

[79] Oliveira A.N., Richards B.J., Slavin M., Hood D.A. (2021). Exercise Is Muscle Mitochondrial Medicine. Exerc. Sport Sci. Rev..

[80] Romanello V., Sandri M. (2021). The connection between the dynamic remodeling of the mitochondrial network and the regulation of muscle mass. Cell. Mol. Life Sci..

[81] Matecki S., Dridi H., Jung B., Saint N., Reiken S.R., Scheuermann V., Mrozek S., Santulli G., Umanskaya A., Petrof B.J. (2016). Leaky ryanodine receptors contribute to diaphragmatic weakness during mechanical ventilation. Proc. Natl. Acad. Sci. USA.

[82] Bertero E., O’Rourke B., Maack C. (2020). Mitochondria Do Not Survive Calcium Overload During Transplantation. Circ. Res..

[83] Cadenas E., Boveris A. (1980). Enhancement of hydrogen peroxide formation by protophores and ionophores in antimycin-supplemented mitochondria. Biochem. J..

[84] Castilho R.F., Kowaltowski A.J., Meinicke A.R., Bechara E.J., Vercesi A.E. (1995). Permeabilization of the inner mitochondrial membrane by Ca2+ ions is stimulated by t-butyl hydroperoxide and mediated by reactive oxygen species generated by mitochondria. Free Radic. Biol. Med..

[85] Kowaltowski A.J., Castilho R.F., Vercesi A.E. (1995). Ca(2+)-induced mitochondrial membrane permeabilization: Role of coenzyme Q redox state. Am. J. Physiol..

[86] Abid H., Ryan Z.C., Delmotte P., Sieck G.C., Lanza I.R. (2020). Extramyocellular interleukin-6 influences skeletal muscle mitochondrial physiology through canonical JAK/STAT signaling pathways. FASEB J..

[87] Smith I.J., Godinez G.L., Singh B.K., McCaughey K.M., Alcantara R.R., Gururaja T., Ho M.S., Nguyen H.N., Friera A.M., White K.A. (2014). Inhibition of Janus kinase signaling during controlled mechanical ventilation prevents ventilation-induced diaphragm dysfunction. FASEB J..

[88] Tang H., Smith I.J., Hussain S.N., Goldberg P., Lee M., Sugiarto S., Godinez G.L., Singh B.K., Payan D.G., Rando T.A. (2015). The JAK-STAT pathway is critical in ventilator-induced diaphragm dysfunction. Mol. Med..

[89] Ferreira L.F., Laitano O. (2016). Regulation of NADPH oxidases in skeletal muscle. Free Radic. Biol. Med..

[90] Powers S.K., Morton A.B., Hyatt H., Hinkley M.J. (2018). The Renin-Angiotensin System and Skeletal Muscle. Exerc. Sport Sci. Rev..

[91] Rosenberg I.H. (1997). Sarcopenia: Origins and clinical relevance. J. Nutr..

[92] Baumgartner R.N., Koehler K.M., Gallagher D., Romero L., Heymsfield S.B., Ross R.R., Garry P.J., Lindeman R.D. (1998). Epidemiology of sarcopenia among the elderly in New Mexico. Am. J. Epidemiol..

[93] Batsis J.A., Mackenzie T.A., Barre L.K., Lopez-Jimenez F., Bartels S.J. (2014). Sarcopenia, sarcopenic obesity and mortality in older adults: Results from the National Health and Nutrition Examination Survey III. Eur. J. Clin. Nutr..

[94] Coen P.M., Musci R.V., Hinkley J.M., Miller B.F. (2018). Mitochondria as a Target for Mitigating Sarcopenia. Front. Physiol..

[95] Franceschi C., Campisi J. (2014). Chronic inflammation (inflammaging) and its potential contribution to age-associated diseases. J. Gerontol. A Biol. Sci. Med. Sci..

[96] Junnila R.K., List E.O., Berryman D.E., Murrey J.W., Kopchick J.J. (2013). The GH/IGF-1 axis in ageing and longevity. Nat. Rev. Endocrinol..

[97] Kwon Y.N., Yoon S.S. (2017). Sarcopenia: Neurological Point of View. J. Bone Metab..

[98] Riuzzi F., Sorci G., Arcuri C., Giambanco I., Bellezza I., Minelli A., Donato R. (2018). Cellular and molecular mechanisms of sarcopenia: The S100B perspective. J. Cachexia Sarcopenia Muscle.

[99] Jackson M.J. (2013). Interactions between reactive oxygen species generated by contractile activity and aging in skeletal muscle?. Antioxid. Redox Signal..

[100] Powers S.K., Jackson M.J. (2008). Exercise-induced oxidative stress: Cellular mechanisms and impact on muscle force production. Physiol. Rev..

[101] Chabi B., Ljubicic V., Menzies K.J., Huang J.H., Saleem A., Hood D.A. (2008). Mitochondrial function and apoptotic susceptibility in aging skeletal muscle. Aging Cell.

[102] Vasilaki A., Mansouri A., Van Remmen H., van der Meulen J.H., Larkin L., Richardson A.G., McArdle A., Faulkner J.A., Jackson M.J. (2006). Free radical generation by skeletal muscle of adult and old mice: Effect of contractile activity. Aging Cell.

[103] Gouspillou G., Bourdel-Marchasson I., Rouland R., Calmettes G., Biran M., Deschodt-Arsac V., Miraux S., Thiaudiere E., Pasdois P., Detaille D. (2014). Mitochondrial energetics is impaired in vivo in aged skeletal muscle. Aging Cell.

[104] Gouspillou G., Sgarioto N., Kapchinsky S., Purves-Smith F., Norris B., Pion C.H., Barbat-Artigas S., Lemieux F., Taivassalo T., Morais J.A. (2014). Increased sensitivity to mitochondrial permeability transition and myonuclear translocation of endonuclease G in atrophied muscle of physically active older humans. FASEB J..

[105] Lee H.C., Chang C.M., Chi C.W. (2010). Somatic mutations of mitochondrial DNA in aging and cancer progression. Ageing Res. Rev..

[106] Picard M., Turnbull D.M. (2013). Linking the metabolic state and mitochondrial DNA in chronic disease, health, and aging. Diabetes.

[107] Short K.R., Bigelow M.L., Kahl J., Singh R., Coenen-Schimke J., Raghavakaimal S., Nair K.S. (2005). Decline in skeletal muscle mitochondrial function with aging in humans. Proc. Natl. Acad. Sci. USA.

[108] Beregi E., Regius O., Huttl T., Gobl Z. (1988). Age-related changes in the skeletal muscle cells. Z Gerontol..

[109] Leduc-Gaudet J.P., Picard M., St-Jean Pelletier F., Sgarioto N., Auger M.J., Vallee J., Robitaille R., St-Pierre D.H., Gouspillou G. (2015). Mitochondrial morphology is altered in atrophied skeletal muscle of aged mice. Oncotarget.

[110] Ibebunjo C., Chick J.M., Kendall T., Eash J.K., Li C., Zhang Y., Vickers C., Wu Z., Clarke B.A., Shi J. (2013). Genomic and proteomic profiling reveals reduced mitochondrial function and disruption of the neuromuscular junction driving rat sarcopenia. Mol. Cell. Biol..

[111] Sebastian D., Sorianello E., Segales J., Irazoki A., Ruiz-Bonilla V., Sala D., Planet E., Berenguer-Llergo A., Munoz J.P., Sanchez-Feutrie M. (2016). Mfn2 deficiency links age-related sarcopenia and impaired autophagy to activation of an adaptive mitophagy pathway. EMBO J..

[112] Tezze C., Romanello V., Desbats M.A., Fadini G.P., Albiero M., Favaro G., Ciciliot S., Soriano M.E., Morbidoni V., Cerqua C. (2017). Age-Associated Loss of OPA1 in Muscle Impacts Muscle Mass, Metabolic Homeostasis, Systemic Inflammation, and Epithelial Senescence. Cell Metab..

[113] Carnio S., LoVerso F., Baraibar M.A., Longa E., Khan M.M., Maffei M., Reischl M., Canepari M., Loefler S., Kern H. (2014). Autophagy impairment in muscle induces neuromuscular junction degeneration and precocious aging. Cell Rep..

[114] Drummond M.J., Addison O., Brunker L., Hopkins P.N., McClain D.A., LaStayo P.C., Marcus R.L. (2014). Downregulation of E3 ubiquitin ligases and mitophagy-related genes in skeletal muscle of physically inactive, frail older women: A cross-sectional comparison. J. Gerontol. A Biol. Sci. Med. Sci..

[115] Joseph A.M., Adhihetty P.J., Wawrzyniak N.R., Wohlgemuth S.E., Picca A., Kujoth G.C., Prolla T.A., Leeuwenburgh C. (2013). Dysregulation of mitochondrial quality control processes contribute to sarcopenia in a mouse model of premature aging. PLoS ONE.

[116] Campbell M.D., Duan J., Samuelson A.T., Gaffrey M.J., Merrihew G.E., Egertson J.D., Wang L., Bammler T.K., Moore R.J., White C.C. (2019). Improving mitochondrial function with SS-31 reverses age-related redox stress and improves exercise tolerance in aged mice. Free Radic. Biol. Med..

[117] Siegel M.P., Kruse S.E., Percival J.M., Goh J., White C.C., Hopkins H.C., Kavanagh T.J., Szeto H.H., Rabinovitch P.S., Marcinek D.J. (2013). Mitochondrial-targeted peptide rapidly improves mitochondrial energetics and skeletal muscle performance in aged mice. Aging Cell.

[118] Singal P.K., Iliskovic N. (1998). Doxorubicin-induced cardiomyopathy. N. Engl. J. Med..

[119] Smuder A.J. (2019). Exercise stimulates beneficial adaptations to diminish doxorubicin-induced cellular toxicity. Am. J. Physiol. Regul. Integr. Comp. Physiol..

[120] Wallace K.B., Sardao V.A., Oliveira P.J. (2020). Mitochondrial Determinants of Doxorubicin-Induced Cardiomyopathy. Circ. Res..

[121] Davies K.J., Doroshow J.H. (1986). Redox cycling of anthracyclines by cardiac mitochondria. I. Anthracycline radical formation by NADH dehydrogenase. J. Biol. Chem..

[122] Doroshow J.H., Davies K.J. (1986). Redox cycling of anthracyclines by cardiac mitochondria. II. Formation of superoxide anion, hydrogen peroxide, and hydroxyl radical. J. Biol. Chem..

[123] Gilliam L.A., Moylan J.S., Patterson E.W., Smith J.D., Wilson A.S., Rabbani Z., Reid M.B. (2012). Doxorubicin acts via mitochondrial ROS to stimulate catabolism in C2C12 myotubes. Am. J. Physiol. Cell Physiol..

[124] Smuder A.J., Kavazis A.N., Min K., Powers S.K. (2011). Exercise protects against doxorubicin-induced markers of autophagy signaling in skeletal muscle. J. Appl. Physiol..

[125] Smuder A.J., Kavazis A.N., Min K., Powers S.K. (2011). Exercise protects against doxorubicin-induced oxidative stress and proteolysis in skeletal muscle. J. Appl. Physiol..

[126] Montalvo R.N., Doerr V., Min K., Szeto H.H., Smuder A.J. (2020). Doxorubicin-induced oxidative stress differentially regulates proteolytic signaling in cardiac and skeletal muscle. Am. J. Physiol. Regul. Integr. Comp. Physiol..

[127] Chandran K., Aggarwal D., Migrino R.Q., Joseph J., McAllister D., Konorev E.A., Antholine W.E., Zielonka J., Srinivasan S., Avadhani N.G. (2009). Doxorubicin inactivates myocardial cytochrome c oxidase in rats: Cardioprotection by Mito-Q. Biophys. J..

[128] Fearon K., Strasser F., Anker S.D., Bosaeus I., Bruera E., Fainsinger R.L., Jatoi A., Loprinzi C., MacDonald N., Mantovani G. (2011). Definition and classification of cancer cachexia: An international consensus. Lancet Oncol..

[129] Sun L., Quan X.Q., Yu S. (2015). An Epidemiological Survey of Cachexia in Advanced Cancer Patients and Analysis on Its Diagnostic and Treatment Status. Nutr. Cancer.

[130] Anker M.S., Holcomb R., Muscaritoli M., von Haehling S., Haverkamp W., Jatoi A., Morley J.E., Strasser F., Landmesser U., Coats A.J.S. (2019). Orphan disease status of cancer cachexia in the USA and in the European Union: A systematic review. J. Cachexia Sarcopenia Muscle.

[131] Vagnildhaug O.M., Balstad T.R., Almberg S.S., Brunelli C., Knudsen A.K., Kaasa S., Thronaes M., Laird B., Solheim T.S. (2018). A cross-sectional study examining the prevalence of cachexia and areas of unmet need in patients with cancer. Support. Care Cancer.

[132] Bozzetti F., Mariani L. (2009). Defining and classifying cancer cachexia: A proposal by the SCRINIO Working Group. J. Parenter. Enter. Nutr..

[133] Dolly A., Dumas J.F., Servais S. (2020). Cancer cachexia and skeletal muscle atrophy in clinical studies: What do we really know?. J. Cachexia Sarcopenia Muscle.

[134] Nosacka R.L., Delitto A.E., Delitto D., Patel R., Judge S.M., Trevino J.G., Judge A.R. (2020). Distinct cachexia profiles in response to human pancreatic tumours in mouse limb and respiratory muscle. J. Cachexia Sarcopenia Muscle.

[135] Johns N., Hatakeyama S., Stephens N.A., Degen M., Degen S., Frieauff W., Lambert C., Ross J.A., Roubenoff R., Glass D.J. (2014). Clinical classification of cancer cachexia: Phenotypic correlates in human skeletal muscle. PLoS ONE.

[136] Zhang Y., Wang J., Wang X., Gao T., Tian H., Zhou D., Zhang L., Li G., Wang X. (2020). The autophagic-lysosomal and ubiquitin proteasome systems are simultaneously activated in the skeletal muscle of gastric cancer patients with cachexia. Am. J. Clin. Nutr..

[137] Judge S.M., Nosacka R.L., Delitto D., Gerber M.H., Cameron M.E., Trevino J.G., Judge A.R. (2018). Skeletal Muscle Fibrosis in Pancreatic Cancer Patients with Respect to Survival. JNCI Cancer Spectr..

[138] Brown J.L., Rosa-Caldwell M.E., Lee D.E., Blackwell T.A., Brown L.A., Perry R.A., Haynie W.S., Hardee J.P., Carson J.A., Wiggs M.P. (2017). Mitochondrial degeneration precedes the development of muscle atrophy in progression of cancer cachexia in tumour-bearing mice. J. Cachexia Sarcopenia Muscle.

[139] Rosa-Caldwell M.E., Benson C.A., Lee D.E., Brown J.L., Washington T.A., Greene N.P., Wiggs M.P. (2020). Mitochondrial Function and Protein Turnover in the Diaphragm are Altered in LLC Tumor Model of Cancer Cachexia. Int. J. Mol. Sci..

[140] Schmitt T.L., Martignoni M.E., Bachmann J., Fechtner K., Friess H., Kinscherf R., Hildebrandt W. (2007). Activity of the Akt-dependent anabolic and catabolic pathways in muscle and liver samples in cancer-related cachexia. J. Mol. Med..

[141] Judge S.M., Wu C.L., Beharry A.W., Roberts B.M., Ferreira L.F., Kandarian S.C., Judge A.R. (2014). Genome-wide identification of FoxO-dependent gene networks in skeletal muscle during C26 cancer cachexia. BMC Cancer.

[142] Padrao A.I., Oliveira P., Vitorino R., Colaco B., Pires M.J., Marquez M., Castellanos E., Neuparth M.J., Teixeira C., Costa C. (2013). Bladder cancer-induced skeletal muscle wasting: Disclosing the role of mitochondria plasticity. Int. J. Biochem. Cell Biol..

[143] Fontes-Oliveira C.C., Busquets S., Toledo M., Penna F., Paz Aylwin M., Sirisi S., Silva A.P., Orpi M., Garcia A., Sette A. (2013). Mitochondrial and sarcoplasmic reticulum abnormalities in cancer cachexia: Altered energetic efficiency?. Biochim. Biophys. Acta.

[144] Shum A.M., Mahendradatta T., Taylor R.J., Painter A.B., Moore M.M., Tsoli M., Tan T.C., Clarke S.J., Robertson G.R., Polly P. (2012). Disruption of MEF2C signaling and loss of sarcomeric and mitochondrial integrity in cancer-induced skeletal muscle wasting. Aging.

[145] de Castro G.S., Simoes E., Lima J., Ortiz-Silva M., Festuccia W.T., Tokeshi F., Alcantara P.S., Otoch J.P., Coletti D., Seelaender M. (2019). Human Cachexia Induces Changes in Mitochondria, Autophagy and Apoptosis in the Skeletal Muscle. Cancers.

[146] White J.P., Baltgalvis K.A., Puppa M.J., Sato S., Baynes J.W., Carson J.A. (2011). Muscle oxidative capacity during IL-6-dependent cancer cachexia. Am. J. Physiol. Regul. Integr. Comp. Physiol..

[147] Marzetti E., Lorenzi M., Landi F., Picca A., Rosa F., Tanganelli F., Galli M., Doglietto G.B., Pacelli F., Cesari M. (2017). Altered mitochondrial quality control signaling in muscle of old gastric cancer patients with cachexia. Exp. Gerontol..

[148] Julienne C.M., Dumas J.F., Goupille C., Pinault M., Berri C., Collin A., Tesseraud S., Couet C., Servais S. (2012). Cancer cachexia is associated with a decrease in skeletal muscle mitochondrial oxidative capacities without alteration of ATP production efficiency. J. Cachexia Sarcopenia Muscle.

[149] Fermoselle C., Garcia-Arumi E., Puig-Vilanova E., Andreu A.L., Urtreger A.J., de Kier Joffe E.D., Tejedor A., Puente-Maestu L., Barreiro E. (2013). Mitochondrial dysfunction and therapeutic approaches in respiratory and limb muscles of cancer cachectic mice. Exp. Physiol..

[150] Mastrocola R., Reffo P., Penna F., Tomasinelli C.E., Boccuzzi G., Baccino F.M., Aragno M., Costelli P. (2008). Muscle wasting in diabetic and in tumor-bearing rats: Role of oxidative stress. Free Radic. Biol. Med..

[151] Fukawa T., Yan-Jiang B.C., Min-Wen J.C., Jun-Hao E.T., Huang D., Qian C.N., Ong P., Li Z., Chen S., Mak S.Y. (2016). Excessive fatty acid oxidation induces muscle atrophy in cancer cachexia. Nat. Med..

[152] Brown J.L., Lawrence M.M., Ahn B., Kneis P., Piekarz K.M., Qaisar R., Ranjit R., Bian J., Pharaoh G., Brown C. (2020). Cancer cachexia in a mouse model of oxidative stress. J. Cachexia Sarcopenia Muscle.

[153] Neyroud D., Nosacka R.L., Judge A.R., Hepple R.T. (2019). Colon 26 adenocarcinoma (C26)-induced cancer cachexia impairs skeletal muscle mitochondrial function and content. J. Muscle Res. Cell Motil..

[154] Wang X., Pickrell A.M., Zimmers T.A., Moraes C.T. (2012). Increase in muscle mitochondrial biogenesis does not prevent muscle loss but increased tumor size in a mouse model of acute cancer-induced cachexia. PLoS ONE.

[155] White J.P., Baynes J.W., Welle S.L., Kostek M.C., Matesic L.E., Sato S., Carson J.A. (2011). The regulation of skeletal muscle protein turnover during the progression of cancer cachexia in the Apc(Min/+) mouse. PLoS ONE.

[156] Rudd K.E., Johnson S.C., Agesa K.M., Shackelford K.A., Tsoi D., Kievlan D.R., Colombara D.V., Ikuta K.S., Kissoon N., Finfer S. (2020). Global, regional, and national sepsis incidence and mortality, 1990–2017: Analysis for the Global Burden of Disease Study. Lancet.

[157] Baldwin C.E., Bersten A.D. (2014). Alterations in respiratory and limb muscle strength and size in patients with sepsis who are mechanically ventilated. Phys. Ther..

[158] Palakshappa J.A., Reilly J.P., Schweickert W.D., Anderson B.J., Khoury V., Shashaty M.G., Fitzgerald D., Forker C., Butler K., Ittner C.A. (2018). Quantitative peripheral muscle ultrasound in sepsis: Muscle area superior to thickness. J. Crit. Care.

[159] Puthucheary Z.A., Rawal J., McPhail M., Connolly B., Ratnayake G., Chan P., Hopkinson N.S., Phadke R., Dew T., Sidhu P.S. (2013). Acute skeletal muscle wasting in critical illness. JAMA.

[160] Jung B., Nougaret S., Conseil M., Coisel Y., Futier E., Chanques G., Molinari N., Lacampagne A., Matecki S., Jaber S. (2014). Sepsis Is Associated with a Preferential Diaphragmatic Atrophy A Critically Ill Patient Study Using Tridimensional Computed Tomography. Anesthesiology.

[161] Iwashyna T.J., Ely E.W., Smith D.M., Langa K.M. (2010). Long-term cognitive impairment and functional disability among survivors of severe sepsis. JAMA.

[162] Brealey D., Brand M., Hargreaves I., Heales S., Land J., Smolenski R., Davies N.A., Cooper C.E., Singer M. (2002). Association between mitochondrial dysfunction and severity and outcome of septic shock. Lancet.

[163] Svistunenko D.A., Davies N., Brealey D., Singer M., Cooper C.E. (2006). Mitochondrial dysfunction in patients with severe sepsis: An EPR interrogation of individual respiratory chain components. Biochim. Biophys. Acta.

[164] Fredriksson K., Rooyackers O. (2007). Mitochondrial function in sepsis: Respiratory versus leg muscle. Crit. Care Med..

[165] Fredriksson K., Hammarqvist F., Strigard K., Hultenby K., Ljungqvist O., Wernerman J., Rooyackers O. (2006). Derangements in mitochondrial metabolism in intercostal and leg muscle of critically ill patients with sepsis-induced multiple organ failure. Am. J. Physiol. Endocrinol. Metab..

[166] Callahan L.A., Supinski G.S. (2005). Sepsis induces diaphragm electron transport chain dysfunction and protein depletion. Am. J. Respir. Crit. Care Med..

[167] Callahan L.A., Supinski G.S. (2005). Downregulation of diaphragm electron transport chain and glycolytic enzyme gene expression in sepsis. J. Appl. Physiol..

[168] Leduc-Gaudet J.P., Mayaki D., Reynaud O., Broering F.E., Chaffer T.J., Hussain S.N.A., Gouspillou G. (2020). Parkin Overexpression Attenuates Sepsis-Induced Muscle Wasting. Cells.

[169] Fredriksson K., Tjader I., Keller P., Petrovic N., Ahlman B., Scheele C., Wernerman J., Timmons J.A., Rooyackers O. (2008). Dysregulation of mitochondrial dynamics and the muscle transcriptome in ICU patients suffering from sepsis induced multiple organ failure. PLoS ONE.

[170] Welty-Wolf K.E., Simonson S.G., Huang Y.C., Fracica P.J., Patterson J.W., Piantadosi C.A. (1996). Ultrastructural changes in skeletal muscle mitochondria in gram-negative sepsis. Shock.

[171] Brealey D., Karyampudi S., Jacques T.S., Novelli M., Stidwill R., Taylor V., Smolenski R.T., Singer M. (2004). Mitochondrial dysfunction in a long-term rodent model of sepsis and organ failure. Am. J. Physiol. Regul. Integr. Comp. Physiol..

[172] Peruchi B.B., Petronilho F., Rojas H.A., Constantino L., Mina F., Vuolo F., Cardoso M.R., Goncalves C.L., Rezin G.T., Streck E.L. (2011). Skeletal muscle electron transport chain dysfunction after sepsis in rats. J. Surg. Res..

[173] Rocheteau P., Chatre L., Briand D., Mebarki M., Jouvion G., Bardon J., Crochemore C., Serrani P., Lecci P.P., Latil M. (2015). Sepsis induces long-term metabolic and mitochondrial muscle stem cell dysfunction amenable by mesenchymal stem cell therapy. Nat. Commun..

[174] Angeras U., Hall-Angeras M., Wagner K.R., James H., Hasselgren P.O., Fischer J.E. (1991). Tissue metabolite levels in different types of skeletal muscle during sepsis. Metabolism.

[175] Supinski G.S., Wang L., Schroder E.A., Callahan L.A.P. (2020). MitoTEMPOL, a mitochondrial targeted antioxidant, prevents sepsis-induced diaphragm dysfunction. Am. J. Physiol. Lung Cell. Mol. Physiol..

[176] Callahan L.A., Supinski G.S. (2007). Diaphragm and cardiac mitochondrial creatine kinases are impaired in sepsis. J. Appl. Physiol..

[177] Callahan L.A., Stofan D.A., Szweda L.I., Nethery D.E., Supinski G.S. (2001). Free radicals alter maximal diaphragmatic mitochondrial oxygen consumption in endotoxin-induced sepsis. Free Radic. Biol. Med..

[178] Javeshghani D., Magder S.A., Barreiro E., Quinn M.T., Hussain S.N. (2002). Molecular characterization of a superoxide-generating NAD(P)H oxidase in the ventilatory muscles. Am. J. Respir. Crit. Care Med..

[179] Clementi E., Brown G.C., Feelisch M., Moncada S. (1998). Persistent inhibition of cell respiration by nitric oxide: Crucial role of S-nitrosylation of mitochondrial complex I and protective action of glutathione. Proc. Natl. Acad. Sci. USA.

[180] Lopez L.C., Escames G., Tapias V., Utrilla P., Leon J., Acuna-Castroviejo D. (2006). Identification of an inducible nitric oxide synthase in diaphragm mitochondria from septic mice: Its relation with mitochondrial dysfunction and prevention by melatonin. Int. J. Biochem. Cell Biol..

[181] Boczkowski J., Lisdero C.L., Lanone S., Samb A., Carreras M.C., Boveris A., Aubier M., Poderoso J.J. (1999). Endogenous peroxynitrite mediates mitochondrial dysfunction in rat diaphragm during endotoxemia. FASEB J..

[182] Supinski G.S., Wang L., Schroder E.A., Callahan L.A.P. (2020). SS31, a mitochondrially targeted antioxidant, prevents sepsis-induced reductions in diaphragm strength and endurance. J. Appl. Physiol..

[183] Mofarrahi M., Sigala I., Guo Y., Godin R., Davis E.C., Petrof B., Sandri M., Burelle Y., Hussain S.N. (2012). Autophagy and skeletal muscles in sepsis. PLoS ONE.

[184] Fletcher S.N., Kennedy D.D., Ghosh I.R., Misra V.P., Kiff K., Coakley J.H., Hinds C.J. (2003). Persistent neuromuscular and neurophysiologic abnormalities in long-term survivors of prolonged critical illness. Crit. Care Med..

